# Removal of Embolus

**Published:** 1914-03

**Authors:** 


REMOVAL OF EMBOLUS. Fritz Bauer, *Zentralblatt für Chir.*, December 20, 1913, reports a successful operation on the abdominal aorta. Key, *Hygeia,* 1913, reported a successful operation for thrombus of the femoral and found one other successful case and two unsuccessful of similar operations on peripheral arteries. Pulmonary embolism has been subjected to operation twelve times in Trendelenberg's clinic and four times in Sauerbruch's, all unsuccessful.

